# Sexual transmission of American trypanosomiasis in humans: a new potential pandemic route for Chagas parasites

**DOI:** 10.1590/0074-02760160538

**Published:** 2017-06

**Authors:** Perla F Araujo, Adriana B Almeida, Carlos F Pimentel, Adriano R Silva, Alessandro Sousa, Sebastião A Valente, Vera C Valente, Manuela M Britto, Ana C Rosa, Rozeneide M Alves, Luciana Hagström, Antonio RL Teixeira

**Affiliations:** 1 Universidade de Brasília, Faculdade de Medicina, Laboratório Multidisciplinar de Pesquisa em Doença de Chagas, Brasília, DF, Brasil; 2 Instituto Evandro Chagas, Belém, PA, Brasil

**Keywords:** Amazon, Trypanosoma cruzi, Chagas disease, family study, humans, epidemiology

## Abstract

**BACKGROUND:**

The *Trypanosoma cruzi* infection endemic in Latin America has now spread to several countries across four continents; this endemic involves triatomine vector-free protists. We hypothesised that the sexual transmission of *T. cruzi* contributes to the ongoing spread of Chagas disease.

**OBJECTIVES:**

A short-term longitudinal study was conducted to evaluate this hypothesis.

**METHODS:**

The study population comprised 109 subjects from four families, among whom 21 had been diagnosed with acute Chagas disease by direct parasitological analysis. Blood mononuclear cells and serum samples were obtained from each study subject once per year for three consecutive years. Enzyme-linked immunosorbent assay (ELISA) and indirect immunofluorescence serological examinations were used to detect specific *T. cruzi* antibodies. Polymerase chain reaction of *T. cruzi* DNA revealed 188-nucleotide bands, which hybridised to a specific radiolabelled probe and were confirmed by cloning and sequencing.

**RESULTS:**

Three independent assessments at different time points revealed *T. cruzi* nuclear DNA footprints in 76% (83/109) of the study population with active infection. In contrast, the ELISA and indirect immunofluorescence assays detected the *T. cruzi* antibody in 28.4% (31/109) of the study samples. Moreover, the semen from 82.6% (19/23) of subjects people revealed harboured the 188- bp base pair *T. cruzi* footprint. Interestingly, the ejaculates of nuclear DNA-positive Chagas patient transmitted the *T. cruzi* upon peritoneal injection or infusion in the vagina of mice, and amastigotes were detected in the skeletal muscle, myocardium, *vas deferens*, and uterine tube.

**MAIN CONCLUSIONS:**

*T. cruzi* infections can be transmitted from females or males to naïve mates through intercourse, and progeny showed discrepancies between the ratios of nuclear DNA footprints and specific antibody that can be explained by the tolerance attained during early embryo growth. Additional studies are needed to develop drugs to eradicate the infections. Additionally, the importance of a vigorous education, information, and communication program to prevent sexually transmitted Chagas disease in humans cannot be underemphasised.

The highly diversified *Trypanosoma cruzi* protozoan of the Order Kinetoplastida and family Trypanosomatidae is important in the medical and veterinary fields (Chagas 1909). American Trypanosomiasis, widely known as Chagas disease, is caused by *T. cruzi* infection and affects mammals. This disease is enzootic from the 42nd parallel north in Northern California down to the 42nd parallel south in the Chubut province of Southern Argentina, where the hematophagous triatomines (Reduviid: Triatominae) bugs agents transmit *T. cruzi* to hundreds of species belonging to eight classes of mammals. Amerindians readily acquired *T. cruzi* from insect vectors that fed on domesticated mammals, and the infections were then passed on to European and African settlers and their descendants (Teixeira et al. 2011a). In the past century, these infections have become hyper-endemic to Latin America and are now present on five continents (Schmunis & Yadon 2010, Albajar-Viñas & Jannin 2011, Pérez-Molina et al. 2012). Acute *T. cruzi* infections are often either asymptomatic or oligosymptomatic and self-limited, but chronically infected people may develop Chagas disease, which is now a leading cause of heart failure in the Western hemisphere. Chagas disease is a clinical condition observed in approximately 30% of people infected with *T. cruzi*; in most cases, the disease attacks the heart (94.5%), but it can also cause megacolon and megaesophagus in the digestive system. Currently, there is no effective treatment for *T. cruzi* infection, and the available treatments for the late manifestations of Chagas disease are unsatisfactory (Lauria-Pires et al. 2000, Teixeira et al. 2011a, Morillo et al. 2015).

The endemic nature of *T. cruzi* infection has been associated with various modes of transmission (Coura et al. 2002). The ancient oral route, insect vector blood-borne infection, blood transfusion, organ transplantation, and accidental transmission in the hospital and research laboratories have contributed to the prevalence of exogenous infections in the human population (Teixeira et al. 2001, Pérez-Molina et al. 2012). Additionally, *T. cruzi* transmission from a mother to her offspring through the placenta is the only currently recognised endogenous source of infection. Murcia et al. (2013) found that transplacental transmission of *T. cruzi* from mothers to offspring occurs in approximately 13.8% of pregnancies.

Early studies (Chagas 1909, Vianna 1911) suggested that *T. cruzi* can spread via sexual transmission. Experimental studies in laboratory animals revealed the presence of *T. cruzi* amastigote nests in the seminiferous tubes, epididymis, and *vas deferens*, as well as in the uterus, tubes, and ovary theca cells of acutely infected mice (Carvalho et al. 2009). Interestingly, the *T. cruzi* life cycle was observed in the ejaculate of a chagasic mouse. Sexual transmission of the parasites from *T. cruzi-*infected mice to naïve recipients was also observed (Alencar et al. 1991). Moreover, amplification of parasite DNA from the germ line cells of infected human hosts and their descendants (Hecht et al. 2010) indicate that *T. cruzi* infections can be sexually transmitted.

Herein, we hypothesized that the endemicity of the infection in urban areas of Latin America, and its recognition to some extent in Africa, Asia, Europe, and Oceania (Schmunis & Yadon 2010, Albajar-Viñas & Jannin 2011, Pérez-Molina et al. 2012), in the absence of insect vectors and of other modes of the infection, can explained by sexual transmission of *T. cruzi*. To determine whether *T. cruzi* can be sexually transmitted in humans, we analysed four families with acute Chagas disease (ACD). Acute cases with fever, headache, malaise, generalised muscle pain, prostration, and occasional signs of heart trouble received medical care. We report a family-based study that reveals the sexual transmission of *T. cruzi* infection in humans.

## SUBJECTS, MATERIALS AND METHODS

*Ethics statement* - The University of Brasilia Medical School Ethical Committee on Human Research approved the research protocol nº 2500.167567/2004-28. Additionally, the Ministry of Health National Commission on Human Research (CONEP Protocol nº 2585/04) in agreement with the National Health Council Resolution 196/1996 determined that all procedures complied with the Brazilian regulations and international guidelines. All adult subjects and all parents or guardians of child participants provided written informed consent on their behalf. The informed consent was signed by adults, parents, or guardians, before collecting blood samples, including positive and negative control samples. These anonymised serum bank samples were maintained at -20ºC. Semen samples were obtained from 23 adult volunteers who signed the informed consent. The free consent form for field studies carried out at the Counties of Barcarena and Breves was approved by the Ethical Committee of the Public Foundation Hospital Gaspar Vianna (protocol nº 054/2009) and by the CONEP/Ministry of Health (protocol nº 11163/2009) related to the study as described previously (protocol nº 2585/2004).

Experiments involving laboratory mice were approved by the Faculty of Medicine Ethical Committee on Animal Research under protocol nº 10411/2011 and were conducted in accordance with the National Council for the Control of Animal Experimentation, guiding line principles for biomedical research involving animals, and Council for International Organization of Medical Sciences and the International Council of Laboratory Animal Sciences.

*Human study population* - This short-run longitudinal study included four families living in the counties of Barcarena and Breves, Pará state, Brazil. The subjects in this family study population comprised 47% females and 53% males ranging in age from 2 to 70 years. Microscopic examination and haemoculture of the blood was conducted to detect *T. cruzi* infections. Of the volunteers in families A (15 individuals), B (44 individuals), C (29 individuals), and D (21 individuals), chronic infections were detected by anti-*T. cruzi* IgG and by parasite nuclear DNA (nDNA) from blood samples (Hecht et al. 2010) obtained at 1, 2, and 3 years after disease onset. Twenty positive controls showed specific *T. cruzi* IgG and nDNA-positive results. Ten negative controls showed the absence of the antibody and nDNA. The research protocol and procedures complied with the Brazilian regulations and international guidelines.

*Parasite growth* - The *T. cruzi* (Chagas 1909) isolate was obtained from Berenice’s blood 55 years after the acute phase of infection from Professor Egler Chiari, Institute of Biology of the Federal University of Minas Gerais, Brazil, and wild-type isolates obtained from the Evandro Chagas Institute (ECI-1 to ECI-21) were grown in the laboratory. Additionally, to confirm that our isolates were *T. cruzi*, we used *Leishmania braziliensis* isolate H3227 from Dr Araujo and Dr Dourado, Institute of Biology of the University of Brasilia. The *T. cruzi* Berenice and wild-type amastigotes and trypomastigotes were maintained in an L6 muscle cell line grown in Dulbecco’s modified Eagle’s medium (DMEM) at pH 7.2 and supplemented with 10% foetal bovine serum, 100 IU/mL penicillin, 100 g/mL streptomycin, 250 nM L-glutamine, and 5% CO_2_ at 37ºC. The *T. cruzi* samples were cultured in blood agar in an axenic liver infusion tryptose medium at 27ºC. The *L. braziliensis* promastigotes were cultured in DMEM supplemented with 20% foetal bovine serum to harvest the parasitic forms during the exponential growth phase. The *T. cruzi* trypomastigotes in the supernatants of the L6 murine cell cultures were used to infect mice and for microscopic examination to search blood trypomastigotes and solid tissue amastigote forms.

*Fluorescence* in situ *hybridisation* - Archetype Berenice *T. cruzi* trypomastigotes and isolates from ACD cases were fixed onto slides before incubation with RNase I (100 µg/µL) for 1 h at 37ºC and then dehydrated with 70%, 90%, and 100% cold ethanol. The parasites were denatured in 50% formamide for 5 min at 92ºC immediately before use, and the 188-nucleotide (nt) probe for nuclear *T. cruzi* DNA was labelled (Molecular Probes, Invitrogen, Carlsbad, CA, USA). The probe (4 ng/µL) was combined with hybridisation buffer supplied with the dye, denatured for 10 min at 72ºC in 50% formamide in 2X SSC, kept on ice for 30 min, and placed onto the slides. The 188-nt DNA probe (10 µL) was mixed with 70 µL of hybridisation buffer and applied to the specimen, and the slide was covered with a coverslip and placed in a humidified chamber at 37ºC overnight. The slide, which was washed for 30 s in 0.4X SSC/0.3% NP-40 at 73ºC, was placed in this solution for 2 min and again washed for 1 min at room temperature by 24ºC in 2X SSC/0.1 NP-40. The primary goat antibody to digoxigenin was identified with an FITC-labelled rabbit anti-goat IgG (Nordic), the slide was examined using an OLYMPUS BX51 light microscope (Tokyo, Japan) with DAPI and FITC filters, and images were collected simultaneously. Additionally, the genotype of the wild-type isolate was determined using the *T. cruzi* Berenice 188-nt digoxigenin-labelled probe, which identified anti-digoxigenin monoclonal antibody-labelled alkaline phosphatase (Molecular Probes). Colour detection was performed using the BCIP and NBT redox system to form a water-insoluble, dark blue precipitate (Teixeira et al. 2001, Mendes et al. 2007, Hecht et al. 2010).

*Nuclear and kinetoplast DNA (kDNA) extraction and purification* - DNA samples extracted from *T. cruzi* epimastigotes, *L. braziliensis* promastigotes, and from venous blood drawn from a cubital vein, and mononuclear cells were separated using a Ficoll-Paque (GE Healthcare, Little Chalfont, UK) gradient (Mendes et al. 2007, Hecht et al. 2010). Sperm samples were suspended immediately in DMEM (1:4 v/v), pH 7.4, and haploid cells collected from the supernatant were incubated for 45 min at 5% CO_2_, 37ºC. Spermatozoa recovered from the supernatant were centrifuged at 13000 x *g* for 5 min and placed in extraction buffer (10 mM Tris-HCL, pH 8.0, containing 10 mM NaCl, 20 mM EDTA, 1% SDS, 0.04% proteinase-K, and 1% DTT) (Moser et al. 1989) prior to DNA extraction. *T. cruzi* kDNA was extracted as described previously (Hecht et al. 2010, Teixeira et al. 2011b). Three independent experiments using human DNA samples were conducted one year apart.

*Primers and probes* - The nDNA primers Tcz1/Tcz2 and kDNA primers S35/S36 (Table) were used for polymerase chain reaction (PCR) amplification (Moser et al. 1989, Sturm et al. 1989) as previously described (Hecht et al. 2010)*.* The probes used in Southern blot hybridisation were: (1) nDNA repetitive sequence (188 bp) obtained by amplification of parasite DNA with the Tcz1/2 primers; (2) wild-type kDNA (∼1.4 kb) minicircle sequences purified from *T. cruzi* epimastigote forms (Hecht et al. 2010). The probes were purified from 1% agarose gels.

**TABLE:** Primers used in the polymerase chain reactions

Primers	DNA template	Sequences
S35	kDNA	5’- ATA ATG TAC GGG (T/G)GA GAT GC -3’
S36	kDNA	5’- GGT TCG ATT GGG GTT GGT G-3’
S34	kDNA	5’- ACA CCA ACC CCA ATC GAA CC- 3’
S67	kDNA	5’- GGTTTTGGGAGGGG(G/C(G/C)(T/G)TC-3’
S35 reverse	kDNA	5’- GCA TCT CMC CCG TAC ATT AT -3’
S67 reverse	kDNA	5’- GAM (G/C(G/C)C CCC TCC CAA AAC C- 3’
L1-1	LINE-1	5’- CTC CGG TCT ACA GTC CCC A- 3’
L1-2	LINE-1	5’- CTC CCA AGA CTA AAC CAG GA- 3’
L1-3	LINE-1	5’- ATC ACA CTC TGG GGA CTG TG- 3’
L1-4	LINE-1	5’- CAC AGT CCC CAG AGT GTG AT- 3’
L1-5	LINE-1	5’- TCC TGG TTT AGT CTT GGG AG- 3’
L16	LINE-1	5’- TGG GAG CTG TAG ACC GGA G- 3’

*Southern blotting and PCR analyses* - Genomic DNA from 109 subjects from four study families, 20 Chagas patients’ positive controls, and 10 uninfected controls were used as templates for PCR with specific *T. cruzi* nDNA Tcz1/2 (Moser et al. 1989) and kDNA primers s35/s36 (Sturm et al. 1989). The standard PCR mixture contained 100 ng template DNA, 0.4 µM of each pair of primers, 2 U Taq DNA polymerase, 0.2 mM dNTPs, and 1.5 mM MgCl_2_ in a 25 µL final volume. The sensitivity of Tcz1/2 primers was determined in a mix of 200 ng chicken DNA with serial dilutions of *T. cruzi* DNA (from 1 ng to 1 fg) and the standard procedure was carried out with the same concentrations of reagents used in the test experiments with human DNA alone (Hecht et al. 2010). The program used was 95ºC for 5 min, followed by 35 cycles at 95ºC/30 s, 64ºC for 1 min, and at 72ºC for 3 min; the reaction was maintained at 72ºC for 5 min and products were stored at 4ºC. The amplification products were analysed on a 1.3% agarose gel and transferred to a positively-charged nylon membrane (GE Life Sciences) using the alkaline method for hybridisation with specific probes labelled with [α-^32^P] dATP using Random Primer Labeling Kit (Invitrogen).

Southern hybridisations were conducted with *Eco*RI (Invitrogen) digests of DNA samples of blood mononuclear cells from uninfected controls, Chagas cases (positive controls), and 109 people belonging to four study families. The enzymes made single cuts in the *T. cruzi* kDNA minicircles, and the digests were subjected to electrophoresis in a 0.8% agarose gel at 50 V overnight at 4ºC. The gel transferred to a positively charged nylon membrane was hybridised with a radiolabelled kDNA or nDNA probe. The membrane was washed twice for 15 min at 65ºC with 2X SSC and 0.1% SDS, twice for 15 min at 65ºC each with 0.2X SSC and 0.1% SDS, and autoradiograph for variable periods of time.

*Amplification of sequences of kDNA minicircles integrated into the human genome* - A modified thermal asymmetric interlaced-PCR (TAIL-PCR) method (Hecht et al. 2010) was used in which kDNA primers were combined with primer sets obtained after aligning chimera sequences (GenBank numbers AAF002199 to AF002203) within the Long Interspersed Nuclear Element (LINE-1) retrotransposon of the human genome (Hecht et al. 2010). In the first round of amplification, each reaction included 200 ng template DNA, 2.5 mM MgCl_2_, 0.4 µM of kDNA primers (S34 or S67), 0.2 mM dNTPs, and 2.5 U *Taq* Platinum (Invitrogen). The kDNA primers were used in combination with 0.04 µM of each LINE-1 (L1-1 to L1-6) primer (Table). Annealing temperatures ranged from 57.9-60.1ºC for kDNA primers, and from 59.9-65.6ºC for LINE-1 primer sets. These temperatures are higher than those (∼45ºC) required for the arbitrary degenerated primers used in TAIL-PCR (Hecht et al. 2010). The temperature and cycles used (MyCycle Thermocycler, Bio-Rad Laboratories, Hercules, CA, USA) have been described previously (Hecht et al. 2010). In the second round of amplification, PCR products were diluted 1:40 (v/v) in water. The kDNA antisense primers S35 and S35 were substituted for the nested primers, along with the same LINE-1 primers. In the third step, PCR products of *tp*TAIL-PCR 2 were diluted 1:10 (v/v) in water and the LINE-1 primers were combined in a reaction with either S67 antisense or S36. PCR products of the final amplification that hybridised with the kDNA probe were cloned directly into the pGEM-T easy vector (Promega, Madison, WI, USA). Clones selected by hybridisation with kDNA probe were sequenced commercially. *tp*TAIL-PCR products were validated in a mix of 300 pg of kDNA from *T. cruzi* with 200 ng of DNA from control subjects who had never been exposed to kDNA. The temperature and amplification cycles were the same as those used for positive control DNA.

*Cloning, sequencing, bioinformatics, and statistical analysis* - The PCR products that hybridised with the repetitive 188-nt *T. cruzi* nDNA and amplification products that hybridised with the kDNA probe were cloned directly into the pGEM T-Easy Vector (Promega). Clones selected by hybridisation with the nDNA and kDNA probe were sequenced commercially (Hecht et al. 2010). CLUSTALW was used for sequence analyses and alignments were performed using BLAST. The expected scores (e-values) were recorded for determining statistical significance (p < 0.001). One-way analysis of variance was used for group analyses, while the Tukey test was employed to compare the means and standard deviations of the experimental data. A p < 0.05 indicated statistical significance.

*Enzyme-linked immunosorbent assay (ELISA*) - The enzyme-linked immunosorbent assay detected *T. cruzi* and *L. braziliensis* soluble antigens (1 µg/100 µL in 0.1 M carbonate buffer, pH 9.6) on coated microplate wells (Mendes et al. 2007). The 1:100 human serum dilutions were incubated with the antigens for 2 h at room temperature. After incubation, the plates were washed three times with phosphate-buffered saline (PBS)/Tween 20 solution before drying, followed by a second incubation (50 μL of a 1:1000 dilution of alkaline phosphatase-conjugated rabbit anti-human IgG, Zymed Laboratories, Inc., Carlsbad, San Francisco CA, USA). After incubation for 90 min at 37ºC, the substrate *p*-nitrophenyl phosphate was added and optical densities (OD) were read at 630 nm in a multi-mode (Bio-Tek Synergy HT, Winooski, VT, USA) plate reader. The test and control sera assays were run in triplicate, and the OD results represent the means ± standard deviation (SD). A serum dilution yielding an absorbance of 0.150 or above was considered a positive reaction. This cut-off was used for bank serum samples. Three independent ELISA experiments were conducted for human serum samples obtained one year apart. For these serological assessments (CONEP protocol n° 2585/2004), we employed de-identified serum bank negative control samples and positive control serum from Chagas patients with parasitological *T. cruzi*.

*Indirect immunofluorescence test (IIF)* - For IIF, 20 µL of human serum dilutions in PBS, pH 7.4, were incubated with glass slides that had been smeared with formalin-killed epimastigotes of *T. cruzi* Berenice or promastigotes of *L. braziliensis*. After 1 h incubation in a moist chamber at 37ºC, the slides were thoroughly washed twice with PBS, air-dried, and treated with a fluorescein-labelled anti-Ig (Zymed Laboratories, Inc., Carlsbad, San Francisco CA, USA). A positive apple-green reaction under the OLYMPUS BX51 microscope with an FITC filter indicated a positive sample in 1:40 and above human serum dilutions (Mendes et al. 2007). IIF assays examined in triplicate in human serum samples obtained one year apart.

*Infection of mice with T. cruzi from Chagas patient semen* - BALB/c mice were originally from the Jackson Laboratory (Bar Harbor, ME, USA) and purchased from the Animal Facilities of the Ribeirão Preto Medical School of São Paulo University. The 12 mice used in these experiments were maintained under positive air pressure at 24ºC and fed Purina chow and water *ad libitum* in our animal room. Aliquots (100 µL) of fresh semen samples from 18-year-old nDNA-positive chronic Chagas patients were intraperitoneally inoculated into three 1-month-old naïve, uninfected, BALB/c mice. Additionally, aliquots of semen (total 100 µL) were infused through a pipette tip into the vagina of three age-matched BALB/c mice. In the control experiments, an equal number of uninfected, one-month-old males were inoculated intraperitoneally with semen from a 20-year-old, nDNA-negative individual, and three females received aliquots of the semen infused into the vagina. Five weeks later, all mice were sacrificed under anaesthesia, and the tissues were fixed in formalin and embedded in paraffin. Serial tissues sections (4-µM thick) were stained with haematoxylin-eosin (H-E).

## RESULTS

**Fig. 1:**
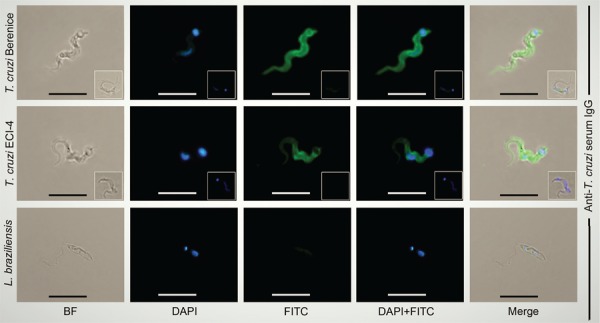
: the phenotype of the *Trypanosoma cruzi* isolates from acutely infected humans. The Berenice protozoan flagellate and the ECI-4 wild-type flagellate shown by bright field (BF) imaging and DAPI (4’, 6-diamidino-2-phenylindole) used to stain parasite kinetoplast (kDNA) and nucleus (nDNA) in blue. These parasite isolates identified with the specific Chagas antiserum IgG that recognizes *T. cruzi* Berenice, ECI-4, but it does not recognize *Leishmania braziliensis* promastigotes after treatment with an FITC-labeled monoclonal antibody to human IgG. The insets show the negative controls. Bars, 20 µM.

In recent decades, physicians in the Brazilian Amazonia (Teixeira et al. 2001, Coura et al. 2002) have faced ACD epidemics. The ACD outbreaks that occurred during 2007 and 2009, with oligosymptomatic cases of acute *T. cruzi* infection, received medical assistance at Gaspar Vianna General Hospital, Belém, Brazil. The ACD patients and their family members volunteered to participate in the research study, which was approved by the ethics committee. These patients from four families lived beside tributaries to the Amazon River in the counties of Barcarena and Breves in the state of Pará, Brazil. Venous blood samples analysed by direct microscopic examination and *in vitro* culture revealed parasite growth. In these families, 21 subjects with some clinical signs and symptoms of acute infection contained the haemoflagellate protozoan in blood cultures as detected at the Evandro Chagas Institute (ECI). Isolates of wild-type protozoa (ECI-1 to ECI-21) were used to infect L6 murine muscle cells and the flagellates that entered the host cells readily appeared as round amastigotes, which continually divided, swarmed the host cells, and burst out into free-swimming trypomastigotes in supernatant medium. Next, 1 × 10^5^ trypomastigotes from the tissue culture supernatant were injected into the peritoneal cavity of mice, and the parasite was detected in the tail blood two weeks later. To further characterise the wild-type protozoan isolates, we subjected the tissue culture trypomastigotes to phenotyping. We used standard antiserum IgG from a Chagas disease case with parasitological demonstration of the haemoflagellate *T. cruzi*. The archetype *T. cruzi* Berenice trypomastigote was a specific target for the Chagas antibody, and the promastigote of its family related to *L. braziliensis*, an agent of cutaneous leishmaniasis, was the negative control (Mendes et al. 2007). We consistently identified ECI-1 to ECI-21 isolates as wild-type *T. cruzi* (Fig. 1).

In this series, each acute infection case received the anti-trypanosome nitro-derivative benznidazole (5 mg/kg of body weight for 60 days per *os*). Human DNA extracted from blood mononuclear cells collected at 1, 2, and 3 years was assessed for the presence of *T. cruzi* nDNA and symbiotic mitochondrion kDNA (Moser et al. 1989, Sturm et al. 1989, Hecht et al. 2010). kDNA, comprising ~30% of the total cellular DNA, contains approximately 20,000 minicircles and a few dozen maxicircles. The *T. cruzi* initiates replication signalling in the symbiotic kinetoplast by relaxation of the minicircle network, which precedes nDNA whole cell division. Despite the critical role of the kinetoplast network in parasite replication, its loss does not result in immediate cell death (Schnaufer et al. 2002). Therefore, demonstration of the nDNA footprint is a unique, essential requirement for the diagnosis of active *T. cruzi* infection.

**Fig. 2:**
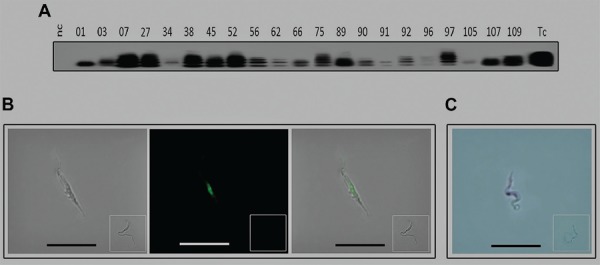
: the *Trypanosoma cruzi* genotype identified in the haemo flagellates from the acute Chagas disease cases. (A) The *T. cruzi* infections identified by the PCR nDNA footprints formed 188 nt bands and catamers after Southern blot with the specific radiolabeled probe; (B) the EC1-4 flagellate identified by the nDNA footprints. The wild-type *T. cruzi* shown by hybridisation with the digoxigenin-labeled 188 nt nDNA probe. From left to right: bright field image; the *T. cruzi* trypomastigote green DNA visible with a primary IgG to the digoxigenin-labeled 188 nt probe after interaction with a secondary fluorescent antibody; and, merge of previous images. The inserts show negative controls; (C) the ECI-10 trypomastigote shown by hybridisation with the Berenice *T. cruzi* 188 nt digoxigenin-labeled probe. The EC1-13 isolate shows a dark nucleus stained with an alkaline phosphatase-labeled monoclonal Ab to digoxigenin. Tc, *T. cruzi*; nc, negative control.

Thus, we probed the DNA collected at three time point from the 21 acutely infected cases using Tc1/2 primer sets that amplify a highly specific repetitive species comprising 7% of total *T. cruzi* nDNA (Moser et al. 1989). We detected protozoan nDNA amplicons in the family members of the ACD cases (Fig. 2A) assessed by three independent experiments conducted with samples obtained one year apart. These amplicons hybridised with the specific radiolabelled 188-nt sequence probe; cloning and sequencing showed that each *T. cruzi* amplicon had the unique 188-nt nuclear repeat motif sequence (Moser et al. 1989). Additionally, protozoan kDNA amplified with primer sets (Sturm et al. 1989) was annealed to minicircle sequences from each of the 21 ACD cases. These results were validated by genotype analysis, whereby a digoxigenin-labelled *T. cruzi* Berenice 188-nt nDNA sequence probe identified homologous repeats in wild-type isolates, which was visualised with a fluorescein-conjugated monoclonal antibody against digoxigenin (Fig. 2B). The specificity of these hybridisation procedures was verified in the negative control *L. braziliensis* promastigotes. An alkaline phosphatase-conjugated monoclonal antibody to the digoxigenin-labelled probe revealed further hybridisation of the digoxigenin-labelled 188-nt sequence with homologous repeats (Moser et al. 1989) in the genomes of wild-type isolates (Fig. 2C). These results show that haemoflagellates in the ACD patients were wild-type *T. cruzi.* The specific 188-bp sequence was present in 20 positive controls and absent in negative controls. Thus, the specific *T. cruzi* nDNA (188-bp) band detected in the 21 benznidazole-treated ACD cases (Fig. 2A) directly demonstrated persistent, chronic infection.

**Fig. 3:**
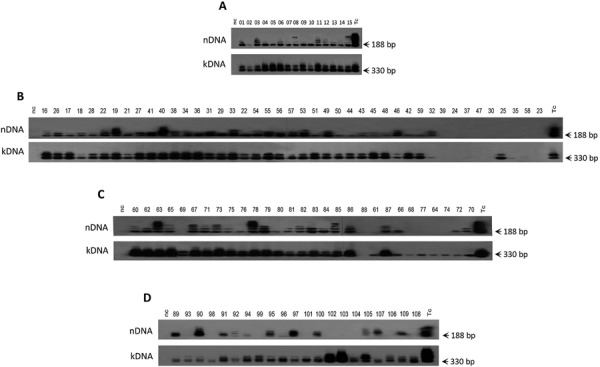
: demonstration of the *Trypanosoma cruzi* nuclear DNA and of its mitochondrial kDNA by Southern blot with the specific radio labeled probes. Family A - *T. cruzi* infections in 15 people, showing positive nDNA and kDNA bands. Family B - In this family, 35 out of and 43 subjects formed the nDNA and kDNA bands. The individual 25 had the kDNA band alone. Family C - Among 29 members of the family C, 22 formed the nDNA and the kDNA bands and five had the kDNA alone. Family D - Eleven out of 21 subjects of the family D formed the nDNA and the kDNA bands; additionally, 10 subjects had the kDNA band alone.

The sensitivity of PCR for nDNA with the Tcz1/2 primer set reached 10 fg, which is 1/24 of the total nDNA (240 fg) from a diploid trypomastigote (Hecht et al. 2010). To evaluate the ratios of *T. cruzi* chronic infections in the study population, we employed the nucleic acid test, which showed high specificity and sensitivity for parasite nDNA footprints in volunteers from the four study families. The specificity and sensitivity of the assay revealed that PCR can be used for high-quality diagnosis of congenital infections and assessment of drug efficacy in Chagas disease chemotherapy (Britto 2009). In these PCR assays, the amplification products that hybridised with the specific radiolabelled 188-nt probe (Moser et al. 1989) formed nDNA bands in 76% (83/109) of the tested samples. Furthermore, the kDNA amplification products formed bands in 92.5% of samples (101/109) tested with specific primer sets (Mendes et al. 2007, Hecht et al. 2010). The results of these experiments are shown in Fig. 3.

**Fig. 4:**
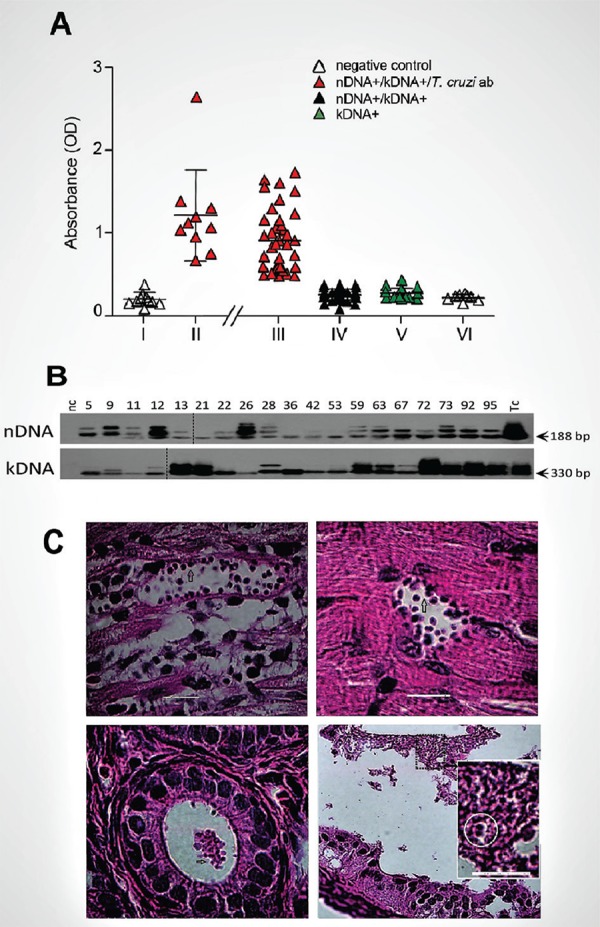
: the *Trypanosoma cruzi* infections demonstrated in the study families. (A) Profiles of *T. cruzi* infections shown by the specific antibody and by the nDNA footprints. Group I (n = 10) and group II (n = 20) were, respectively, the negative and the positive control antiserum to *T. cruzi* infections. Group III split subgroups: III-a (n = 31) families’ cases with the infections confirmed by nDNA-positive and specific antibodies to *T. cruzi*; III-b (n = 52) included families with the infections detected by the *T. cruzi* nDNA-positive in the absence of specific antibodies; III-c (n = 18) included families displaying kDNA-positive only in the absence of nDNA and of specific antibodies; III-d (n = 8) included the study population individuals with all tests negative. (B) Profiles of the nDNA and kDNA present in the germ cell line. Nineteen out of 23 semen samples contained the *T. cruzi* infections shown by the positive nDNA and kDNA assays. Tc, *T. cruzi*; nc, negative control. (C) The infectivity of the *T. cruzi* from human ejaculate inoculated in mice. Left, *T. cruzi* amastigotes nest in the heart (top) and in the lumen of the *vas deferens* (bottom). Right, *T. cruzi* amastigotes nest in the skeletal muscle (top) and in the uterine tube (bottom). The insert shows a dividing amastigote (circle). Bars, 25 µM. Arrows show amastigote parasites.

The ELISA and IIF tests detect serum *T. cruzi* antibodies (Mendes et al. 2007). These assessments consistently detected specific *T. cruzi* antibodies in 28.4% (31/109) of the study population. Eight individuals (7.4%) remained free of *T. cruzi* antibodies, nDNA, and kDNA. The plots of ODs obtained from the ELISAs for the 109 individuals and serum-bank positive and negative controls are shown in (Fig. 4A). The negative and positive control, respectively, group I and II serum dilutions revealed optical density (ODs) low readings significantly different (p < 0.05) from those high readings of the test group III for breaking open in subgroups IIId to IIIa (Fig. 4A). Thus, there were broad discrepancies between the presence of specific *T. cruzi* antibodies and PCR test results: 37.4% (31/83) of patients yielded both positive nucleic acids and specific antibody assays (III-a), whereas 62.6% (52/83) showed nDNA+ and kDNA+ bands in the absence of specific antibodies (III-b). Eighteen subjects (16.5%) showed integrated kDNA alone (III-c), while eight people (7.4%) had none (III-d).

The 1960 Nobel Prize winners, Sir Frank McFarlane Burnet and Peter Brian Medawar explained that difference depending on the immune tolerance acquisition during early embryo with immature immune system.

To clarify this point, we isolated *T. cruzi* nDNA from the germ cell line of subjects at their reproductive ages. Haploid DNA revealed *T. cruzi* nDNA+- and kDNA+-specific bands in 19 of 23 (82.6%) semen samples (Fig. 4B). Additionally, the infectivity of *T. cruzi* from a Chagas patient’s ejaculate, which tested positive in nDNA assays and lacked specific antibodies, was demonstrated by injecting 100 µL of semen into the peritoneal cavity of male mice and through infusion of an equal amount of semen into the vagina of 1-month-old female BALB/c mice. Five weeks later, we detected *T. cruzi* amastigote nests in the heart and skeletal muscles, and clumps of differentiating parasites were present in the lumen of the *vas deferens* and uterine tube (Fig. 4C). These markers were not detected in control mice that received nDNA-negative semen.

**Fig. 5:**
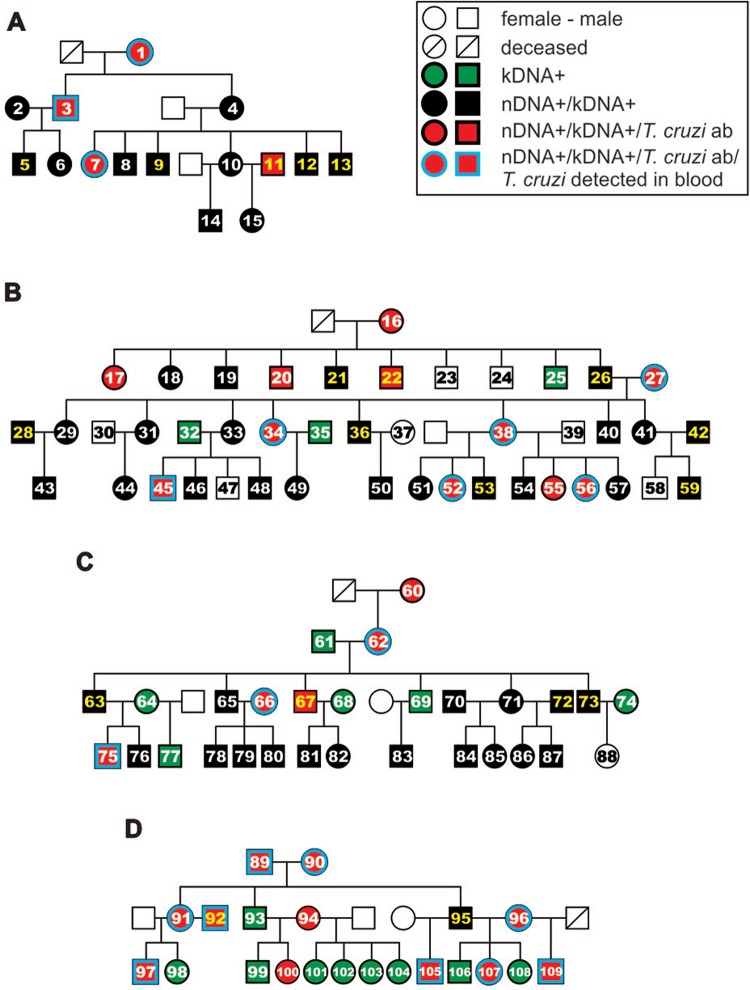
: heredograms and personalised profiles of the *Trypanosoma cruzi* infections in four study families. Family A - of the 15 members tested, four had the *T. cruzi* IgG antibody, nDNA and kDNA; 11 had the parasite nDNA and kDNA, and five (yellow numbers) had *T. cruzi* in their ejaculates. Family B - of the 44 samples, 11 had the *T. cruzi* IgG antibody, nDNA and kDNA and 23 had the parasite nDNA and kDNA footprints in the absence of antibody. In this family, *T. cruzi* nDNA found in the ejaculates of seven subjects: three members had kDNA alone integrated into the genome, and seven had none of the above (numbered white circles and squares). Family C: - of the 29 members tested, five had the *T. cruzi* IgG antibody, nDNA and kDNA, 17 had the parasite nDNA and kDNA footprints, six had integrated kDNA only and one patient had none of the above. In this family four members had the *T. cruzi* active infections (yellow numbers) in the ejaculates; Family D: - of the 21 subjects tested, 11 had the *T. cruzi* IgG, nDNA and kDNA; one had the nDNA and kDNA, nine members had integrated kDNA only; two subjects (92 and 95) had the *T. cruzi* active infections in the ejaculates.

Heredograms with personalised profiles enabled rapid visualisation of study family data. Fig. 5 shows family subjects with infections in the presence of both IgG anti *T. cruzi* and its nDNA and infections in the absence of IgG but in the presence of parasite nDNA. Additionally, 18 subjects (16.5%) without active *T. cruzi* infection contained kDNA minicircle alone integrated into the human genome. This finding can be explained by the insertion of mutations into specific *loci* in the human genome (EMBL HG008116 to HG008708) either through active *T. cruzi*-infection or through kDNA inheritance from the parents (Hecht et al. 2010, Teixeira et al. 2011b, Guimaro et al. 2014).

## DISCUSSION

In this study, we present epidemiological, clinical, parasitological, immunological, and nucleic acid data indicating that human *T. cruzi* infections can be sexually transmitted. We assessed active *T. cruzi* infections in the somatic and in the germ cell line of individuals in a family study population. We documented the parasite nDNA footprints in the blood nucleated cells and in the haploid cells of the semen ejaculates, as well as in the absence of the specific *T. cruzi* antibody in the serum of the chagasic patients.

The parasitological demonstration of the protozoan in 21 ACD cases was fundamental to our covalidation of the PCR nDNA footprints, which formed specific 188-nt bands in the samples from *T. cruzi-*infected subjects only. The diagnosis made using this standard laboratory assay was compared with the results of specific immunological tests. The broad differences between the ratios of positive results obtained with parasite-specific antibodies (37.4%) and those with nDNA tests (62.6%) indicated that most cases, in the absence of specific antibodies, stem from sexually transmitted *T. cruzi* infections during embryo development before maturation of the immune system. This agrees with the demonstration of *T. cruzi* nDNA footprints in the semen of 19 of 23 chagasic males in the absence of specific serum antibodies in the study families.

The symbiotic kDNA is a landmark of the kinetoplastid protozoan, but the PCR assay with the symbiont DNA primer sets (Sturm et al. 1989) should not be used to diagnose Chagas disease because the kDNA sequence alone integrates into the host genome and is inherited by the progeny (Hecht et al. 2010, Teixeira et al. 2011b, Guimaro et al. 2014); we detected the *T. cruzi* kDNA minicircle sequences alone integrated into the host genome in 16.5% (18/109) of family members without active infection. However, these findings suggest a potential for the development of genetically driven inflammatory lesions, and the immune tolerance status was correlated with the outcome of autoimmune disease over time (Teixeira et al. 2011b, Guimaro et al. 2014).

Immune tolerance in chickens hatched from *T. cruzi*-inoculated eggs in the absence of the specific antibody was observed after challenge with the formalin-killed flagellate (Guimaro et al. 2014). Herein, tolerance is a natural phenomenon resulting from altered immune system immunoglobulin expression and abrogation of the functional signalling of self-reactive B lymphocytes (Poncini et al. 2008), which explains the absence of the specific *T. cruzi* antibody in patients with the nDNA-positive 188-nt band (Hecht et al. 2010). Additionally, immunologic self-tolerance in *T. cruzi* infections is associated with regulatory dendritic cells *in vitro* (Poncini et al. 2008); tolerance was maintained by CD25^+^/CD25^+^ fox3-positive T cells (Belkaid 2008) expressing IL-2 receptors α-chains (Sakaguchi et al. 1995). The development of full tolerance requires *T. cruzi* infection at an early embryo age with an immature immune system, which recognises the parasite thereafter as a component of its own body (Teixeira et al. 2011b, Guimaro et al. 2014).

Interestingly, infusions into the vagina or intraperitoneal inoculation of the semen from Chagas patients showing the nDNA footprint, but without the specific *T. cruzi* antibody, generated nests of *T. cruzi* amastigotes in the heart, skeletal muscles, *vas deferens*, and uterine tube of mice five weeks later. The *T. cruzi* amastigote clumps and transforming trypomastigote detected in reproductive organs, which were confirmed by immunoperoxidase assays.

Furthermore, in a mouse model system, da Silva (2013) found that *T. cruzi* infections were transmitted to naïve mates during intercourse. These experiments showed that healthy, control female or male mice that sexually mated with a *T. cruzi*-infected male or female acquired the flagellate. Crossbreeding of males or females showing the nDNA footprints to naïve females or males generated progeny with active *T. cruzi* infections. Breeding of *T. cruzi*-infected mice with naïve mates generated progeny showing nDNA footprints in 58.6% (41/70) of samples examined, but the specific antibodies were present in as few as 22% (9/40) of mice progeny (da Silva 2013). Furthermore, nests of *T. cruzi* amastigotes were documented in the reproductive organs of the progeny.

The experimental findings in the mouse model indicate that *T. cruzi* can be sexually transmitted in humans. Moreover, in the absence of a demonstrable port of entry of exogenous *T. cruzi,* either through the skin or other route of infection, we conclude that a minority of Chagas family members showed the specific antibody because the sexually transmitted infectious agent reached a foetus with a mature immune system. The *T. cruzi*-infected females and males sexually transmit the infectious agent to uninfected, naïve mates during intercourse and, additionally, the progeny retain active infection in the semen or uterine secretions into the vagina (da Silva 2013).

The potential epidemiologic dispersion of infections by the sexual route correlates with the persistence of *T. cruzi* forms in the male and female organs of reproduction. In this study, clumps of replicative *T. cruzi* forms in the sexual organs of mice associated local immune privilege as described elsewhere (Wood & Sakaguchi 2003, Niederkorn 2006, Fujisak et al. 2011), thus explaining why the sexual transmission is common. The observed ratios of sexual transmission of *T. cruzi* infections may require reproduction systems to achieve sustainable immune privilege that bolsters parasite growth. The immune privilege described for the testicles, uterus, tubes, and ovary may explain the uncurbed growth of *T. cruzi* in reproductive organs (Bonomo & Savino 2015) such that the parasite can reach a sexual partner through uterine secretions and ejaculates. Immune privilege is a phenomenon that allows some organs (testes, uterus, eyes, and brain) to down-regulate inflammatory reactions to avoid damage to sensitive and specific functions (Wood & Sakaguchi 2003, Niederkorn 2006). Hormones (Fujisak et al. 2011) and several immune factors down-regulate macrophages, natural killer cells, T lymphocytes, and regulatory Treg cells, thus orchestrating the inhibition of some pro-inflammatory cytokines and immune privilege triggers (Wood & Sakaguchi 2003, Fujisak et al. 2011). Moreover, this study showed that *T. cruzi* has a unique survival capability, such that it continuously adapts to a non-hostile environment from which it can escape before the host dies. In this regard, *T. cruzi* is a sexually transmitted mutagenic agent that promotes evolution, descendants with modifications, and natural selection (Hecht et al. 2010, Teixeira et al. 2011b, Guimaro et al. 2014).

In conclusion, examination of chagasic families enabled us to combine clinical with experimental data to reveal the sexual transmission of *T. cruzi* infections. Thus, the dispersion of human Chagas disease to five continents (Schmunis & Yadon 2010, Albajar-Viñas & Jannin 2011, Pérez-Molina et al. 2012) is possible. Additional studies needed to clarify whether Chagas disease-free parallels socially equal popopulation, and Chagas disease would never threaten this far (Hotez et al. 2012).

Further family studies are needed to map the prevalence of *T. cruzi* infections, likely acquired sexually and through various other routes, by human populations in different ecosystems. Moreover, the development of a drug without genotoxic effects is essential to eradicate *T. cruzi* infections and prevent Chagas disease.
